# In vitro characterization of *Trichophyton rubrum* biofilm by combined anti-biofilm enzymes

**DOI:** 10.1371/journal.pone.0331291

**Published:** 2025-09-10

**Authors:** Amna Rehman, Fatima Ismail, Ayesha Safdar, Muhammad Imran

**Affiliations:** 1 Department of Biochemistry, Institute of Biochemistry, Biotechnology and Bioinformatics, The Islamia University of Bahawalpur, Bahawalpur, Pakistan; 2 The Islamia University of Bahawalpur, Bahawalpur, Pakistan; 3 Shenzhen University Institute for Advanced Study, Shenzhen, Guangdong, China; Dow University of Health Sciences, PAKISTAN

## Abstract

*Trichophyton rubrum*, a dermatophyte, demonstrates a notable ability to form mature biofilms on skin and associated surfaces, strengthening its resistance to antifungal agents. This characteristic poses intricate challenges in dermatological research and therapeutic strategies, underscoring the need for innovative approaches to effectively manage fungal infections. This work assessed the impact of the anti-biofilm enzymes, i.e., cellulase, protease, and amylase, individually and in combination, on the eradication and inhibition of *T. rubrum* biofilm. After 168 hours of incubation, *T. rubrum* biofilm formation matured, and the anti-biofilm enzymes significantly reduced the rate of biofilm development. The rates of biofilm inhibition and eradication for cellulase, protease, and amylase were 64%, 38%, and 28% at 72 hours of incubation, and 47%, 25.87%, and 17%, respectively, at 168 hours. However, the combined anti-biofilm enzymes (cellulase, protease, and amylase) had a 60.62% biofilm suppression rate. SEM analysis revealed marked reductions in *T. rubrum* conidial density, disrupted hyphal structures, and diminished biofilm adherence in enzyme-treated samples compared to untreated controls, visually supporting the inhibitory effect observed in quantitative assays. These morphological alterations indicate compromised fungal viability and structural disintegration of the biofilm matrix. Collectively, the SEM findings reinforce the therapeutic potential of enzyme-based strategies against dermatophytic biofilms. Additionally, these anti-biofilm enzymes have shown strong efficacy in reducing the exopolysaccharide (EPS) content and degrading the complex EPS matrix network. The *T. rubrum* biofilm treated with anti-biofilm enzymes, such as cellulase and protease, resulted in EPS degradation by FTIR analysis. The antibiofilm enzyme cellulase showed notable degradation of the beta 1–4 linkage within the glycosidic bond. A significant degradation is observed when *T. rubrum* biofilm is treated with combined enzymes (cellulase, protease, and amylase). The combined enzymatic treatment disrupted the EPS matrix, indicating its potential as an effective strategy for inhibiting biofilm formation by T. rubrum.

## 1. Introduction

The pathogenic keratinolytic fungi known as dermatophytes are the cause of dermatophytosis, an illness that affects both people and animals [1]. Fungal infections that are superficial and affect the skin and its appendages are called dermatomycoses [2]. Even though dermatophytic infections are usually superficial, they can sometimes spread, especially in immunocompromised people, and are obstinately challenging to treat. For example, poor nail penetration by antifungal drugs and recurrent recurrence despite prolonged treatment make onychomycosis (tinea unguium) a severe therapeutic problem [3]. *Trichophyton rubrum* is the primary cause of athlete’s foot (tinea pedis), an anthropophilic dermatophyte with strict host specificity for humans. Because of its tenacity and recurrence, it poses serious issues in clinical dermatology [4]. Accurate and timely diagnosis is crucial for effective dermatophytosis therapy; balancing molecular approaches benefits against traditional methods limitations. Choosing molecular diagnostic techniques involves factors like time, cost, technical complexity, species coverage, and the required diagnostic yield. The applicability of molecular methods depends on the mycology lab’s background and resources, creating a tailored decision-making framework for the specific diagnostic needs [5].

The ability of dermatophytes to create biofilm, a virulence feature that increases antifungal resistance, is a noteworthy recent finding in the field of dermatophytosis [6]. Treatment for dermatophytes is challenging due to their diverse defense mechanisms against antifungal stress. Medication efflux and biofilm formation are two important processes that contribute to drug tolerance; cellular efflux is the outcome of coordinated management of efflux pumps [7]. According to recent research, phage-derived enzymatic cocktails may be a useful tactic for breaking through the protective extracellular polymeric substances (EPS) barrier in fungal biofilms. To target persistent microbial communities, multienzyme formulations that break down proteins, polysaccharides, and signaling molecules have showed promise. Growing antimicrobial resistance is driving the use of these enzyme-based strategies in industrial and biomedical settings [8]. Anti-biofilm enzymes are considered effective and eco-friendly agents for biofilm control, as they can break down the extracellular matrix and facilitate detachment. Enzymes such as amylase and proteinase effectively eliminate biofilms, while cellulase can reduce biomass and microcolony development, allowing for targeted action based on the specific components of the extracellular matrix present in different microorganisms [9].

*Trichophyton rubrum* biofilm was tested for suppression and elimination by anti-biofilm enzymes (cellulase, proteinase, and amylase). The growth of the biofilm was significantly hindered by the anti-biofilm enzymes, and the biofilm content of *T. rubrum* reached its peak after 120 hours of incubation. Cellulase, protease, and amylase showed a 64%, 38%, and 28% inhibition and eradication rate in the biofilm after 72 hours of incubation; however, by 120 hours, those same rates were 47%, 25.87%, and 17%, respectively. FTIR analysis showed that protease enzymatically broke down the proteins, whereas cellulase exhibited substantial degradation of the beta 1–4 linkage inside the glycosidic bond. The *T. rubrum* biofilm was denser and generated more biomass and EPS. Recent SEM-based analyses confirmed the presence of dense hyphal networks and conidial structures embedded within extracellular polymeric substances in T. rubrum and T. mentagrophytes biofilms, underscoring the structural complexity that contributes to antifungal resistance and highlighting potential targets for novel antifungal therapies [10]. It can also help with future adjustments to the dosage and length of therapy for antifungals that are presently on the market [11]. SEM micrographs showed that only individual conidia clung to the nail surface for up to 96 hours, according to *T. rubrum* micrographs, and after 168 hours, a few genuine hyphae started to grow along the whole preparation extension. The *T. rubrum* single biofilm showed solitary conidia at 24 and 96 hours, and some genuine hyphae were seen after 168 hours [12].

Despite the increasing incidence of dermatophytic infections, the formation of biofilms by *Trycophyton rubrum* has made treatment more challenging due to enhanced resistance to antifungal agents and host immune defenses. While chemical antifungals are commonly used, their efficacy is often limited against biofilm-embedded cells, and prolonged use can lead to side effects or resistance development [13]. Although anti-biofilm enzymes have shown potential in disrupting microbial biofilms in bacterial and yeast models, their application against dermatophyte biofilms remains largely underexplored [14]. To date, there is limited literature assessing the effect of individual and combined enzyme treatments on different developmental stages of T.rubrum biofilms. Although recent studies [13] have evaluated the effect of proteinase k on mature dermatophyte biofilms, investigation on the use of other enzymes remain limited. This paucity of research motivated for the examination of multienzyme strategies targeting different biofilm matrix component.

The increasingly clinical concern of *T.rubrum* biofilm-associated infections and the limitations of conventional antifungal therapies, the present study aimed to investigate the inhibitory potential of anti-biofilm enzymes individually and in combination on the biofilm formation of *Trycophyton rubrum*. The objectives were to evaluate their efficacy at both premature and mature stages of biofilm development through crystal violet staining assay for biomass quantification, scanning electron microscopy (SEM) for morphological characterization and FTIR spectroscopy for biochemical assessment of the biofilm matrix.

Although enzyme-based strategies for bacterial biofilm disruption have been studied [9,15], their application against fungal biofilms—particularly those formed by *Trichophyton rubrum*—remains underexplored. Furthermore, the time-course development, structural composition, and enzyme-based eradication strategies for *T. rubrum* biofilms are not well understood. Therefore, this study was designed to: (1) evaluate the temporal progression of *T. rubrum* biofilm formation; (2) characterize biofilm architecture using FTIR and SEM analyses; and (3) assess the biofilm-inhibitory and eradication efficacy of cellulase, amylase, and protease enzymes. Our aims offer new insights into enzyme-based antifungal strategies targeting dermatophytic biofilms.

## 2. Materials and methods

### 2.1. Isolate and growth conditions

The strain of *Trichophyton rubrum* MN691068.1 was obtained from the Department of Biochemistry, The Islamia University of Bahawalpur, Pakistan [16] and cultured on SDA (Sabouraud dextrose agar) medium. SDA plates were made with 40, 10, 20, and 0.5 g/L of dextrose, peptone, agar, and chloramphenicol to inhibit the unwanted microbial contamination, respectively. The plates cultured with strain were incubated in an incubator (Velp Scientifica, model FTC 90E, Usmate, Italy) at 30 ºC for about 7–15 days.

Fungal biofilms were incubated every day while being cultured at 30°C for a maximum of 15 days. Based on visual markers, such as the development of a consistent, dense fungal mat and pigmentation on the reverse side of the culture plate, which varied from yellowish-brown to wine-crimson, biofilm maturity was assessed. When starting downstream experiments, these traits were the standard criterion because they were consistently seen around day 14.

### 2.2. Qualitative tube assay

To assess biofilm formation, a qualitative assay was performed, as reported by [17]. Freshly grown culture isolates were inoculated in sterile test tubes containing 10 mL of tryptic soy broth and incubated for 7 days for dermatophytes at 37 ºC. After complete incubation, media was poured out of the tubes, washed with phosphorous buffered saline (PBS), and dried at room temperature. Then, 0.1% crystal violet was used to stain the dried test tubes. The excess stain was removed with distilled water, and the tubes were dried again. All the experiments were repeated three times.

All quantitative data were analyzed using Microsoft Excel. Results are expressed as mean ± standard deviation (SD) of three independent experiments (n = 3). For the time-course biofilm formation assay, one-way analysis of variance (ANOVA) was performed to assess differences between time points. A p-value < 0.05 was considered statistically significant. In cases where only one control OD value was available, statistical comparison was limited to descriptive statistics and percentage inhibition was calculated accordingly.

A sterile, uninoculated broth was included as a negative control to confirm that crystal violet staining and ring formation were due to fungal biofilm and not due to media components or non-specific binding.

### 2.3. Biofilm formation assay

A 96-well plate method, modified from [18], was used to evaluate early biofilm development. Dermatophyte isolates were inoculated on Sabouraud Dextrose Agar (SDA) and incubated at 28°C for 7 days to promote sporulation. The harvested spores were suspended in 5 mL phosphate-buffered saline (PBS), vortexed, and filtered. The spore concentration was adjusted to 1 × 10⁶ CFU/mL using a hemocytometer, and validated by viable colony counts on SDA. After that, 100 µL of the standardized suspension was inoculated into wells of a sterile, flat-bottom 96-well microtiter plate and incubated at 30°C for 3 hours to allow pre-adhesion. Wells were then gently washed with PBS to remove non-adherent cells, and 200 µL of RPMI 1640 medium (with L-glutamine, without bicarbonate) buffered to pH 7.0 with MOPS was added. Plates were incubated at 30°C for 72 hours. The time-course experiment was conducted with three biological replicates (n = 3), and each time point measurement was performed in triplicate.

RPMI 1640 was selected for its consistent nutrient profile and its proven suitability for dermatophyte biofilm formation and antifungal testing, as reported in previous literature.

Each time point included a positive control with T. rubrum culture grown in RPMI, and a negative control consisting of sterile RPMI medium without fungal inoculation.

### 2.4. Quantification of biofilm by crystal violet staining

The biofilm biomass was quantified using the procedure outlined by [2]. The culture media was taken out of the pre-formed mature biofilms on micro-titer plates, adherent cells were triple-washed in PBS, and the wells were allowed to air dry for twenty minutes. Next, each well was filled with 0.5% crystal violet in a volume equal to 100 µL of solution, and the staining process was allowed to run for 15 minutes. After de-staining the wells with distilled water, 100 µL of pure ethanol solution was used to wash them in order to completely dissipate the crystal violet. Next, 200 µL of the solution was moved to a fresh micro titer plate, and the plate was read using an ELISA reader set at 570 nm for the remaining fungal isolates [19]. All the experiments were performed in triplicate. The growth of biofilm in a micro-titer plate was assessed using the optical density (OD) of fungal biofilm isolates and compared to an OD control (ODC). The formula provided was used to determine biofilm formation [20].


OD≤ODC=non−adODC=non−adherent

ODC<OD≤2ODC=weaklyadherent

2ODC<OD≤4ODC=moderatelyadherent

4ODC<OD=stronglyadherent

For *Trichophyton rubrum*, the inoculation is done at different time intervals, i.e., 2 h, 24 h, 72 h, 96 h etc. to predict at which incubation time the biofilm development is highest.

### 2.5. Analysis of the bioﬁlm formation and eradication of *T. rubrum*

To determine the final concentration of the enzyme solution for testing, 200 µL of the inoculum and enzymes were added in a 1:3 ratio to 96-well microtiter plates with lids. The microtiter plates were incubated for 72, 96, and 120 hours at 30°C with shaking (180 rpm). After incubation, the broths were carefully pipetted out, and the planktonic cells with loose attachments were extracted. The biofilms stuck to the edges and bottom of the microtiter plate were washed with 1 milliliter of sterile distilled water. Using the previously mentioned crystal violet staining technique, the quantity of biofilm was quantified. The biofilm formation rate (%) is calculated as follows: biofilm of treatment (OD 570 nm)/ biofilm of control (OD 595 nm) ×100. The control group comprised of 200 µL of RPMI media having 7.2–7.4, conducted on the same microtiter plates. There were three biological replicates and three technological replicates.


$$Thebiofilmformationrate(\%)=\frac{\text{Biofilmoftreatment}(\text{OD570nm})}{\text{Biofilmofcontrol}(\text{OD595nm})}\times \text{100}$$

In order to analyze the biofilm eradication rate, *T. rubrum* cell suspensions (1.0 × 10^6^ CFU/mL) in microtiter plates were incubated at 30 ºC with 180 rpm shaking. The precipitates were removed from the broths and rinsed with 1 milliliter of sterile distilled water before 300 µL of enzymes, i.e., cellulase produced by *T. rubrum* using the substrate, i.e., treated wheat, protease, and amylase produced from *Candida tropicalis* in the Biochemistry Laboratory of The Islamia University of Bahawalpur, were added separately and then in combination. After the plates were incubated at 30°C for 72 and 120 hours, the liquids were drained, and the contents of the biofilm were examined. The bioﬁlm eradication rate (%) = (bioﬁlm of control (OD 570 nm)-bioﬁlm of treatment (OD 595 nm)/bioﬁlm of control (OD 570 nm) ×100. The effects of individual enzymes and combined enzymes on *T. rubrum* biofilm eradication were assessed after 72 and 168 hours of incubation [9].


$$Thebiofilmeradicationrate(\%)=\frac{\text{Biofilmofcontrol}(\text{OD570nm})-\text{Biofilmoftreatment}(\text{OD595nm})}{\text{Biofilmofcontrol}(\text{OD595nm})}\times \text{100}$$

For each enzyme assay, untreated wells with fungal biofilm served as positive controls, while buffer-only treated wells were used as negative controls to validate that the observed biofilm reduction was enzyme-specific.

### 2.6. Statistical analysis

All quantitative data were analyzed using Microsoft Excel. Results are expressed as mean ± standard deviation (SD) of three independent experiments (n = 3). For the time-course biofilm formation assay, one-way analysis of variance (ANOVA) was performed to assess differences between time points. A p-value < 0.05 was considered statistically significant. In cases where only one control OD value was available, statistical comparison was limited to descriptive statistics and percentage inhibition was calculated accordingly.

### 2.7. Observation of bioﬁlm by SEM

The enzyme and fungal cell suspensions were added to the test tube in a 1:3 ratio, and the enzyme solution’s final concentration was determined to be the test concentration. Next, a sterile glass sheet measuring 2 cm by 1 cm by 2 mm was placed within the test tube. The equivalent volume of buffer solution was added to the control group. For 72 hours, the test tubes were incubated at 30°C with 180 rpm of shaking in the shaking incubator. The glass sheet was washed with PBS to remove non-adherent cells and air-dried under sterile conditions. The dried slides were then sent to an external facility for SEM processing and imaging9. At the facility, samples were *sputter-coated with gold* and imaged using a *JEOL JSM-6490 scanning electron microscope* operated at an accelerating voltage of 20 kV and magnification of 1000 × . Image metadata including scale, working distance, and electron signal type are provided in the supplementary file. This method allowed for qualitative assessment of biofilm morphology across treatment groups.

Untreated *T.rubrum* biofilm served as positive controls to visualize typical hyphal networks and biofilm matrix, while enzyme-treated samples were compared for structural alterations. Sterile glass sheet without fungal inoculation acted as negative control.

### 2.8. FTIR spectroscopy

The *Trichophyton rubrum* stain is inoculated in an autoclaved culture tube containing TSB, i.e., about 100 microliters in 5 mL of the tryptic soya broth. This is incubated for about 5 days at 30°C in a shaking incubator. After incubation, the media was discarded, and the biofilm was washed three times in order to remove or wash the planktonic cells from the biomass. The biomass adhered to the walls of the culture tube was separated, dried, and analyzed under an FTIR spectrometer. For the treatment of enzymes, i.e., cellulase, protease, and combined enzymes (cellulase, protease, and amylase), the above procedure was repeated until incubation. After that, the media was discarded carefully so that the biofilm remained intact. 4–5 mL of enzyme was added, i.e., cellulase, to the culture tube and was placed in the shaking incubator for 3–4 h. After that, the supernatant was discarded and washed three times to remove the planktonic cells. The biomass adhered to the wall was separated, air dried, and analyzed on the FTIR spectrometer. The process was repeated again for protease and combined enzymes [21].

FTIR spectra of untreated fungal biofilms were used as positive controls to represent normal biochemical profiles, whereas spectra of enzyme-treated samples were analyzed to detect chemical bond alterations. Sterile samples served as negative references when needed.

## 3. Results

### 3.1. Growth of *Trichophyton rubrum* on SDA

**Fig 1:**
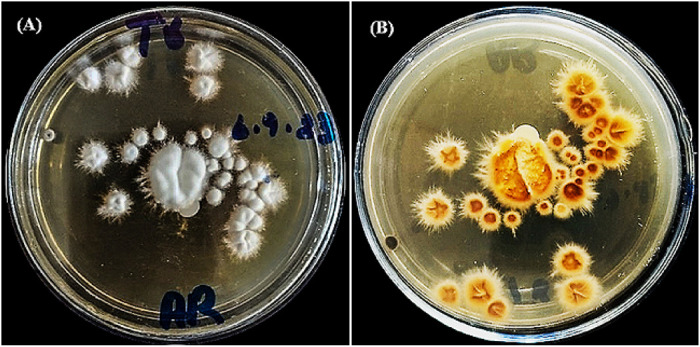
Growth of the *Trichophyton rubrum* at different time intervals, i.e., 7^th^ and 14^th^ days. (a) frontal view and (b) reverse view.

The culture of *Trichophyton rubrum* MN691068.1 was inoculated on Sabouraud dextrose agar SDA. The texture of the culture surface ranged from downy to suede-like. The pigmentation on the culture surface varied from white to deep red. The pigmentation on the reverse side of the culture can differ from yellowish-brown to wine crimson. Fig 1 clearly demonstrates the frontal and reverse views of the *Trichophyton rubrum* (Tr 55) at the 7^th^ and 14^th^ days.

### 3.2. Qualitative tube method

**Fig 2:**
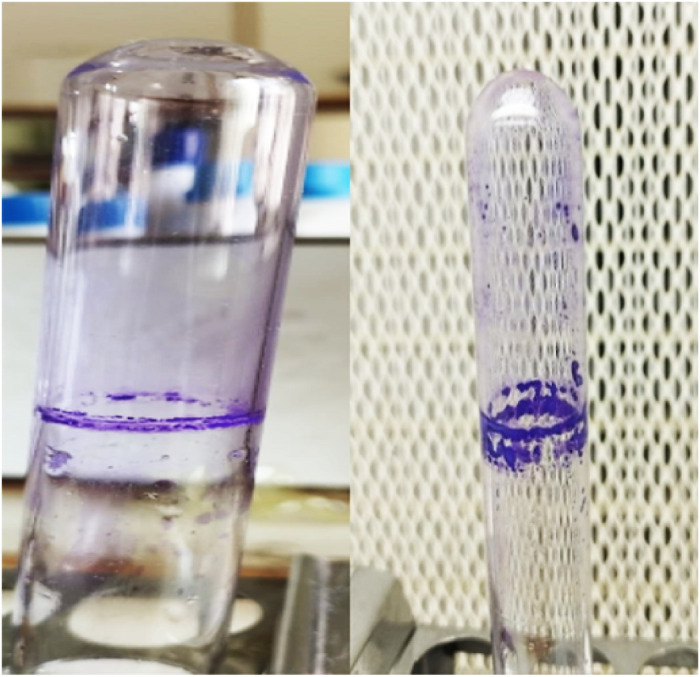
Biofilm rings develop on the bottom surface and walls of test tubes. *Trichophyton rubrum* is a strong biofilm producer.

The fungal isolate had biofilm developed around the test tube’s bottom surface and walls in the shape of a ring. Clear biofilm growth is shown by a definite line around the test tube’s sides and bottom. This is used to assess the growth characteristics of *Trichophyton rubrum* in the tube over a designated incubation period. This includes the visible changes in the medium. These changes included biofilm ring formation around the tube walls and at the bottom of the tube, as shown in Fig 2, which indicated that the formation of *T. rubrum* differs in terms of incubation time. At 3 days, the ring formed around the tube wall is weak as compared to that formed at 7 days. Fig 2, shown below, indicates that *T. rubrum* is a strong biofilm producer.

Sterile uninoculated tubes containing tryptic soy broth were used as negative controls to ensure that the biofilm ring formation was attributable to fungal growth and not media residues. Whereas, positive control consisted of a test tube inoculated with *T. rubrum in broth medium.

### 3.3. Quantitative analysis (time course study)

The results revealed that in the case of *T. rubrum*, the growth of biofilm is gradually increasing for up to 7 days. The results reveal a significant increase in the optical density (OD) at 570 nm within a 7-day timeframe. This trend aligns with the observed temporal dynamics, emphasizing a prolonged and substantial growth in biofilm density.

Biofilm biomass increased progressively from 12 h to 168 h, as measured by OD at 570 nm. One-way ANOVA showed a highly significant difference among time points (p = 8.79 × 10 ⁻ ¹¹). The OD reached its maximum at 168 h, indicating mature biofilm formation. Statistical significance was observed at multiple time points compared to the 12 h baseline, and is indicated in the figure by asterisks (*p < 0.05, **p < 0.01, ***p < 0.001).

**Fig 3:**
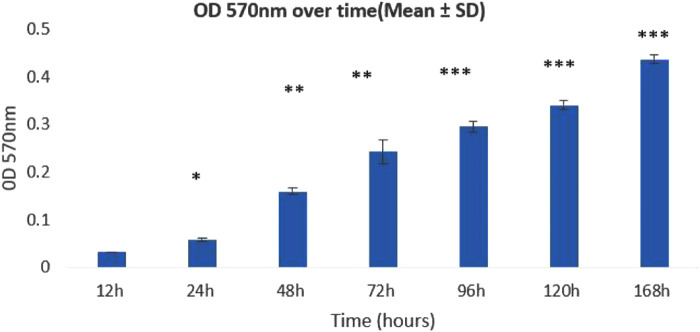
The graphical represents the time-course of biofilm formation by Trichophyton at various intervals of time. The OD value at 570 nm examined the increase in biofilm formation consistently after every 12 hours until 168 hours. Bars represent mean ± SD of triplicate experiments. Statistical analysis was conducted using one-way ANOVA with post-hoc comparison to the 12 h baseline. p < 0.05, **p < 0.01, ***p < 0.001 vs. 12 maximum biofilm biomass was observed at 168 h.

The quantification assay revealed a significant increase in optical density at 570 nm over a period of 7 days, indicating robust and accelerated *T. rubrum* biofilm development. This accelerated growth pattern indicates the quick establishment of a dense fungal biofilm within the timeframe that was observed, highlighting the dynamic nature of biofilm formation. These results provide important new understandings of the temporal dynamics of *T. rubrum* biofilm, illuminating its rapid maturation as shown in Fig 3.

### 3.4. Enzyme impact on *T. rubrum* biofilm formation and eradication

**Fig 4:**
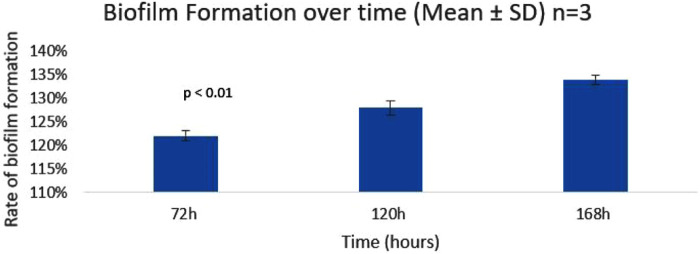
Rate of biofilm formation over time (72h, 120h, 168h) expressed as percentage. Bars represent mean ± SD (n = 3). Statistical significance across time points determined via one-way ANOVA (p = 8.79 × 10 ⁻ ¹¹).

Fig 4 shows that the rate of biofilm formation increases as the incubation time increases. The percentage in the rate of biofilm formation of *T. rubrum* increased until 168 h. The rate of biofilm formation for *T. rubrum* is the highest at 168 h as compared to 72 h because at this stage the biofilm becomes mature.

Among the enzymes tested, cellulase demonstrated the greatest reduction in biofilm biomass, with a mean inhibition rate of 47%, followed by protease (25.87%) and amylase (17%). These values were calculated based on OD readings at 570 nm using the standard inhibition formula. Although each control was represented by a single OD value, the treated groups were tested in triplicate, showing consistent inhibitory trends. The absence of control replicates limited statistical analysis with control groups, and this limitation is acknowledged.

**Fig 5:**
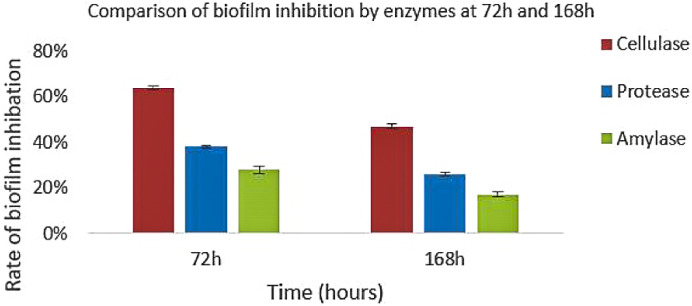
Comparative effect of cellulase, protease, and amylase on biofilm inhibition at 72 h and 168 h. The bar graph illustrates the percentage inhibition of Trichophyton rubrum biofilm formation following treatment with cellulase, protease, and amylase enzymes at two time points: 72 hours and 168 hours. Error bars represent the standard deviation (±SD) from three independent replicates (n = 3). The combined presentation allows for direct comparison of enzyme efficacy over time.

**Fig 6:**
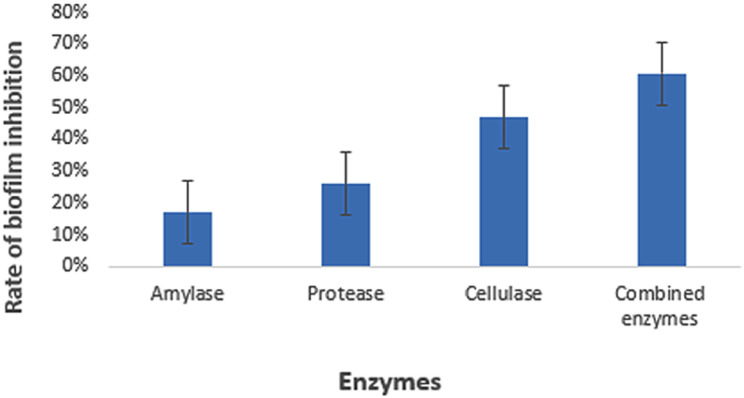
Comparison between the rate of *T. rubrum* biofilm inhibition between the individual enzymes and the combined anti-biofilm at 168h.

Figs 5 and 6 show that the rate of inhibition of biofilm by anti-biofilm enzymes is highest in the case of cellulase as compared to protease, which in turn has a higher inhibition rate as compared to amylase. The rate of biofilm inhibition is highest in the case of combined enzymes (cellulase, protease, and amylase) as compared to individual enzymes. According to the data given above, it is clearly noticed that the rate of inhibition of biofilm is highest for cellulase, protease, and amylase at 72 h rather than at 168 h, because at 168 h the biofilm is at its maturation stage and the cells are tightly bound to the walls, so it’s difficult to degrade them from the walls as compared to the cells of the biofilm at 72 h. The inhibitory effects of the anti-biofilm enzymes varied according to the stages of biofilm development, with inhibition rates of 64%, 47% at 72 h, and 168 h in the case of cellulase, respectively. The inhibition rate was highest at 168 h for combined enzymes. Fig 8 shows the comparison in the inhibition rate of *T. rubrum* biofilm at 168 h by the action of both combined anti-biofilm enzymes (cellulase, protease, and amylase) and anti-biofilm enzymes.

**Fig 7:**
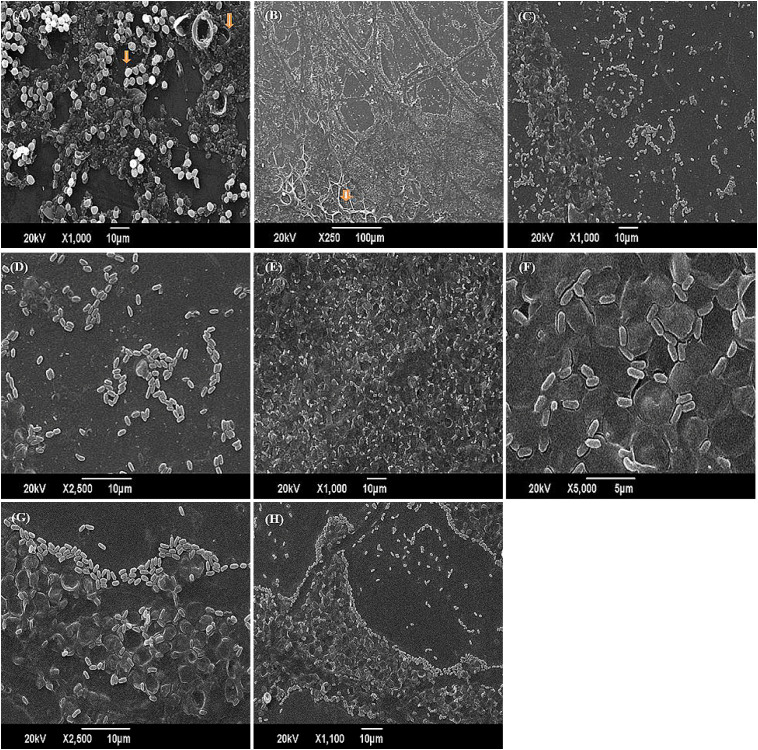
(A) is the SEM image of *T.rubrum* biofilm at 1000× magnification presents a clear view of the conidial elements embedded in the extracellular biofilm matrix. The image highlights the dense clustering of round fungal cells, typical of mature biofilm architecture. The conidia and matrix are labelled by (B) is the SEM image of *T.rubrum* biofilm (at 250 × magnification) provides an overview of the filamentous hyphae, which are prominently observed in the lower-left region, forming an interwoven network. These hyphal structures are represented by an arrow. (c) is the SEM image of *T.rubrum* biofilm treated with cellulase at 1000 × magnification, residual extracellular material and scattered conidia indicate partial biofilm matrix degradation. (D) is the also the SEM image of *T.rubrum* biofilm treated with cellulase at 2500 × magnification, individual conidia are visible with no evident hyphal structures, suggesting that cellulase disrupted the biofilm integrity*, leading to dispersal of fungal cells. Scale bar = 10 µm. (E) is the SEM image of *T.rubrum* biofilm treated with Protease at 1,000x magnification, the fungal surface appears disrupted and lacks distinct hyphal networks, indicating possible degradation of surface structures. (F) is the SEM image of *T.rubrum* biofilm treated with Protease at 5,000x magnification, fragmented and deformed fungal elements are visible, likely representing damaged hyphae or remnants of the fungal biofilm matrix, consistent with proteolytic activity. (G) and H are the SEM image of *T.rubrum* biofilm treated with Amylase at 2500 × magnification, clustered conidia remain embedded in *intact biofilm matrix*, especially in the lower half.

### 3.5. Observation of bioﬁlm by scanning electron microscopy (SEM)

SEM scan revealed that the fungi were covered in a matrix of biofilm, and following treatment with various enzymes, very few fungal cells stuck to the board. With well-developed conidia and undeveloped hyphae, it is evident from these images that the *T. rubrum* biofilm is in the pre-adhesion phase. After several anti-biofilm enzyme treatments, these pictures showed that the biofilm matrix had been disturbed and that the majority of the biofilm cells had separated from it. Because of their compact and intricate structure, the biofilm cells were extremely resistant to stress conditions and might hinder the penetration of antimicrobials and access to the deep-positioned cells. The matrix that forms the basis of the three-dimensional structure constituted the bioﬁlm and accounted for more than 90% of the dry mass of a bioﬁlm. Consequently, degradation of the matrix is an effective method for deracinating microbial bioﬁlm. The use of enzymes is a direct anti-bioﬁlm approach. Previous studies have shown that some single enzymes can effectively inhibit the bioﬁlm. According to the studies, the cellulase enzymes could degrade the biofilm matrix more than protease and amylase.

Fig 7. Scanning electron micrographs of biofilms on glass surfaces before and after enzyme treatment. Samples were air-dried and gold-coated before imaging at 1000 × magnification using JEOL JSM-6490 SEM at 20 kV. These images provide qualitative evidence of structural disruption compared to untreated controls.

The SEM image of untreated *Trichophyton rubrum* biofilm in figure A and B at 1000 × magnification showing abundant conidia (Bright white or light grey,round) embedded in an extracellular matrix(darker gray, amorphous spread over background). Scale bar = 10 µm. SEM image of untreated T. rubrum biofilm at 250 × magnification. Filamentous hyphae are clearly visible, forming a network within the biofilm matrix in case of 250 × magnification.

The SEM image at 1000 × magnification of *T.rubrum* biofilm treated with cellulase in figure C and D shows oval bodies are most likely conidia. The biofilm matrix appears disrupted or collapsed, especially when compared to untreated images. There is a clear reduction in cell clustering, suggesting enzymatic damage to extracellular polysaccharides. At 2500 × magnification, the Conidia remain intact, though spread out and in small clumps. Cellulase effectively disrupted the cellulose components of the biofilm matrix.

The SEM images of protease in figure E and F treatment caused degradation of fungal surface structures, as evidenced by the collapsed or fragmented appearance of the hyphae and absence of intact conidia in treated *T. rubrum* samples.

The SEM images in figure G and H of *T.rubrum* biofilm amylase shows a thick layer of biofilm is still present at the bottom half. Conidia (oval-shaped spores) are abundant and grouped, especially along the *upper central ridge. Matrix appears less degraded compared to cellulase/protease treatments. Clustering suggests limited disruption.

### 3.6. FTIR spectroscopy

**Fig 8:**
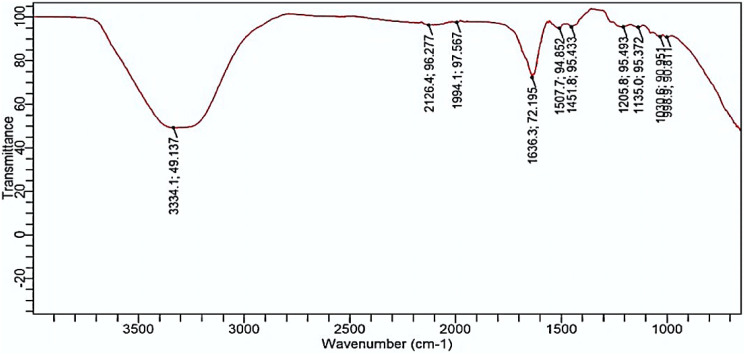
FTIR spectrum of *T. rubrum* biofilm (untreated biofilm).

Fig 8 shows the control (untreated *T. rubrum* biofilm). The FTIR spectra of the untreated biofilm (control) show various peaks indicating the presence of certain biological macromolecules: peak around 1205.79 cm^-1^ (intensity 94.85) may indicate carbon-oxygen bonds in carbohydrates and esters; peak around 1451.80 cm^-1^ (intensity 95.43) indicates carbon-hydrogen bonds in fatty acids and lipids; peak at 1507.71 cm^-1^ (intensity 94.85) indicates nitrogen-hydrogen bonds in amides; and peak at 1636.30 cm^-1^ (intensity 72.19) indicates carbon-oxygen double bonds in amides.

**Fig 9:**
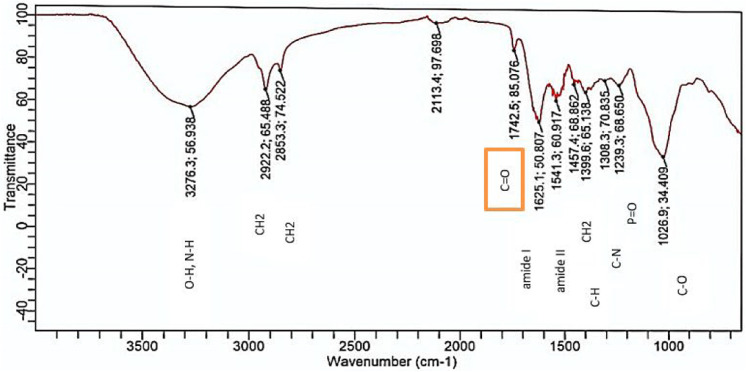
FTIR spectrum of *T. rubrum* biofilm treated with cellulase.

Fig 9 shows the FTIR spectra of *T. rubrum* biofilm treated with cellulase. The FTIR spectra of *T. rubrum* biofilm after cellulase treatment show changes indicating the breakdown of carbohydrate bonds. The C-O stretching peak (carbohydrates) shifts and decreases in intensity with enzyme treatment, especially with cellulase, showing the breakdown of carbohydrate structures. The control has a peak at 1205.79 cm^-1^ (intensity 94.85), whereas the biofilm treated with cellulase showed a peak at 1026–1030 cm^-1^ (intensity 61.64). The reduction in intensity and slight shift of the peak around 1026–1030 cm^-1^ suggest the breakdown of beta 1–4 glycosidic linkages in cellulase.

**Fig 10:**
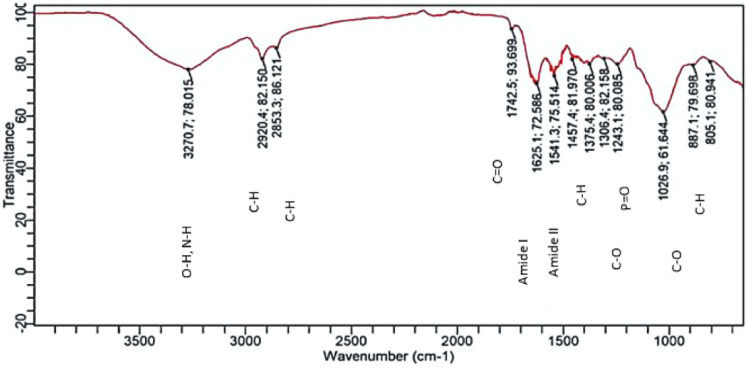
FTIR spectrum of *T. rubrum* biofilm treated with protease.

Fig 10 shows the FTIR spectra of *T. rubrum* biofilm after treatment with protease. The FTIR spectra highlight changes in protein structures. The peptide bonds in proteins are at a wavenumber of 1625 cm^-1^ (amide I band, C = O stretching) and 1541 cm^-1^ (amide II band, N-H bending, and C-N stretching). The intensity of amide I and amide II decreased and slightly shifted in position with enzyme treatments, including enzyme degradation. The control has amide I and amide II at wavenumbers 1507.71 cm^-1^ and 1636.30 cm^-1^ (intensity 94.85 and 72.19), whereas the biofilm treated with protease has wavenumbers 154 cm^-1^ and 1625 cm^-1^ (intensity 60.73 and 50.83). It was observed that the reduced intensity in the amide I and II regions indicated the hydrolysis of peptide bonds.

**Fig 11:**
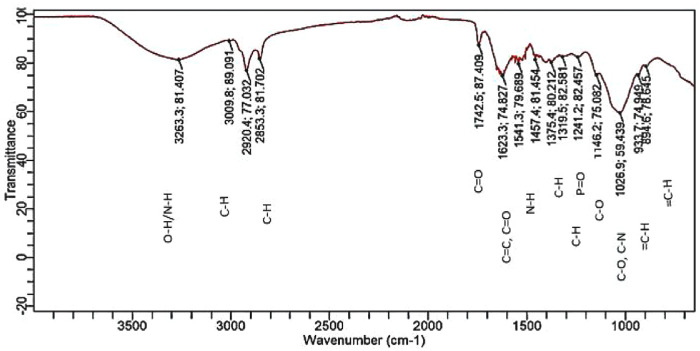
FTIR spectrum of *T. rubrum* biofilm treated with combined enzymes (cellulase, protease, and amylase).

Fig 11 shows the FTIR spectra of *T. rubrum* biofilm treated with combined enzymes (cellulase, protease, and amylase). The comparison between the spectra shows certain changes that involve shifts in the peaks and decreases in the intensity as mentioned in Table 1. In the case of proteins, the control peak is at 1507.71 cm^-1^ and 1636.30 cm^-1^ (intensity 94.85 and 72.19), whereas the biofilm treated with combined enzymes showed peaks at 1541.25 cm^-1^ and 1623.26 cm^-1^ (intensity 79.68 and 74.82). In the case of carbohydrates (C-O), the untreated biofilm peaked at 1205.79 cm^-1^ (intensity 95.49), and the biofilm treated with combined enzymes showed peaks at 1026.88 cm^-1^ (intensity 59.43). For lipids (CH-2 bending and C = O stretching peaks), untreated biofilm peaks at 1451.80 cm^-1^ (intensity 95.43), and biofilm treated with combined anti-biofilm enzymes peaks at 1457.39 cm^-1^ and 1742.53 cm^-1^ (intensity 81.45 and 87.70), respectively. The significant changes in peaks at 1026.88 cm^-1^ and the appearance of new peaks at 933.70 cm^-1^ and 894.56 cm^-1^ suggest extensive degradation of glycosidic linkages, including both alpha and beta linkages.

**Table 1:** Comparative FTIR Spectral Analysis of untreated *T.rubrum* biofilm and Enzyme treated *T.rubrum* biofilm.

Functional Group/ Assignment	Untreated (cm ⁻ ¹)	Treated Biofilm (cm ⁻ ¹)	Bond Shift/Change	Enzyme Applied	Interpretation/ observation
C–O stretch (carbohydrates)	1205.79 (intensity 94.85)	1026-1030 (intensity 61.64)	Downshift	Cellulase	Glycosidic bond cleavage; β-1,4 linkage degradation
Amide I (C = O stretch, proteins)	1636.3 (intensity 72.19)	1625 (intensity 50.83)	Slight downshift	Protease	Peptide bond hydrolysis; protein denaturation
Amide II (N–H bend)	1507.7 (intensity 94.85)	1541 (intensity 60.73)	Upward shift	Protease	Partial hydrolysis of peptide bonds in structural proteins Shift suggests alteration in protein conformation
C–O stretch (carbohydrates)	1205.79 (intensity 94.85)	1026.88 (intensity 59.43)	Downshift	Combined enzymes	Enzymatic synergism leading to enhanced breakdown of extracellular polysaccharides
Amide I (C = O stretch	1636.3 (intensity 72.19)	1623.26 (intensity 74.82)	Downshift	Combined enzymes	Intensified peptide bond cleavage and protein denaturation
Amide II (N–H bending)	1507.7 (intensity 94.85)	1541.25 (intensity 79.68)	Upward shift	Combined enzymes	Strong conformational change in protein due to combined enzyme action
O-H/N-H stretch	3334.1	3263.28	Shifted lower	Combined enzymes	Hydrogen bonding disrupted; protein matrix loosening
Ester C = O stretching (Lipids)	1451.80 (intensity 95.43)	1742.53 (intensity: 87.70)	New peak Appearance	Combined enzymes	Indicates lipid degradation and possible breakdown of membrane-bound lipids or release of esterified compounds
New glycosidic peaks	_	933.70, 894.56	New peak Appearance	Combined enzymes	Suggest cleavage of β-1,6 or α linkage in complex carbohydrates

## 5. Discussion

According to recent metabolic and phylogenomic research, *T. rubrum* can exhibit significant phenotypic variation in response to environmental cues, even if its genome is primarily clonal. After laser irradiation, Zhang et al. (2023) [22] showed notable changes in metabolic phenotype (such as nutrition usage profiles), suggesting that phenotypic plasticity can happen even in the absence of genetic alteration. According to Ohst [23], phenotypic variability is more likely to be the result of regulatory or epigenetic dynamics than of genotype variations. Due to variations in temperature, humidity, pH, and nutritional composition, the *T. rubrum* strain exhibits a broad spectrum of surface textures and coloring. As we can clearly see from our results, *T. rubrum* has a downy to suede-like appearance, whereas it has a reverse pigmentation of yellowish-brown to crimson red. Crystal violet and safranin staining data from earlier research demonstrate that *T. rubrum* produces more biomass in its biofilm than *T. mentagrophytes* [13]. The study found that *T. rubrum* biofilm can be quantified in three hours, with significant growth by 48 hours and peak development at 72 hours. Over seven days, the biofilm density increased steadily, reaching an optical density (OD) greater than 0.437 at 570 nm. These findings highlight the rapid and dynamic growth of *T. rubrum* biofilms, which may inform antifungal strategies. The most frequent fungus that causes dermatophytosis in people is *Trichophyton rubrum*. Keratinized structures, such as skin, hair, and nails, are invaded by dermatophytes. Except in certain immunosuppressed patients, *T. rubrum* infections are often restricted to the superficial skin and are frequently chronic and recurrent [24]. In current research anti-biofilm enzymes to break down the exopolysaccharide in *T. rubrum* biofilm in order to treat the disease’s recurrent and chronic symptoms and assess how well they worked. This methodology duplicates an earlier investigation utilizing an alternative bacterial strain [25]. According to this previous study, lipase, cellulase, and proteinase K worked together to disrupt the EPS matrix and lower the exopolysaccharide content, which effectively reduced and destroyed the foodborne pathogen V. parahaemolyticus biofilm. The enzymes offered a viable remedy for the food sector by removing mature biofilm and inhibiting all stages of biofilm production [26]. In current research, the anti-biofilm enzymes used were cellulase, protease, and amylase, both individually and in combination. The cellulase is produced from *T. rubrum* by solid-state fermentation using treated wheat bran as a substrate. Whereas protease and amylase are produced from *Candida tropicalas* by solid-state fermentation using rice bran and wheat as substrates, respectively. The enzymes cellulase from produced from Trichophyton rubrum and amylase and protease from *Candida tropicalis* were validated for activity using standard protocols: the FPase for cellulase, the tyrosine liberation assay for protease and DNSA assay for amylase. Enzyme activity was quantified in µmol/min based on standard curves prior to their experimental application, ensuring consistency across treatments.

According to the results, it is evident that *T. rubrum* biofilm formation rises with incubation time. Especially at 72 h, cellulase shows the strongest biofilm inhibition compared to 168 h, when biofilms are more developed and difficult to break. The inhibitory effects of the anti-biofilm enzymes differed depending on the stage of biofilm development; for cellulase, protease, and amylase, the inhibition rates were 64%, 38%, and 28% at 72 h, and at 168 h, they were 47%, 25%, and 18%, respectively. Thus according to the previous study, the use antibiofilm enzymes either alone or in combination have proved to be efficient in the eradication of microbial biofilm efficiently [27].

In comparison to the prior study, the current study show growth of the biofilm was significantly hindered by the anti-biofilm enzymes, and the biofilm content of T. rubrum reached its peak after 120 hours of incubation. Cellulase, protease, and amylase showed a 64%, 38%, and 28% inhibition and eradication rate in the biofilm after 72 hours of incubation; however, by 168 hours, those same rates were 47%, 25.87%, and 18%, respectively. FTIR analysis showed that protease enzymatically broke down the proteins, whereas cellulase exhibited substantial degradation of the beta 1–4 linkage inside the glycosidic bond. The *T. rubrum* biofilm was denser and generated more biomass and EPS. The SEM images showed microconidia and a well-organized network of hyphae oriented in every direction, partially embedded in EPS. Studying and characterizing the biofilms that dermatophytes create can help find novel medications to treat these mycoses [10]. It can also help with future adjustments to the dosage and length of therapy for antifungals that are presently on the market [11]. SEM micrographs showed that only individual conidia clung to the nail surface for up to 96 hours, according to T. rubrum micrographs, and after 168 hours, a few genuine hyphae started to grow along the whole preparation extension. The T. rubrum single biofilm showed solitary conidia at 24 and 96 hours, and some genuine hyphae were seen after 168 hours [12].

Studies highlighted the effectiveness of enzymatic treatment in dispersing established medical biofilms, emphasizing the role of EPS-degrading enzymes as promising adjuncts to antimicrobial therapy. This supports our findings, where such enzymes disrupted the biofilm of *T.rubrum*, enhancing potential treatment strategies [28]. Prior studies have demonstrated that a well-organized extracellular matrix is essential for the formation of biofilms, as exemplified by the *T. rubrum* biofilm, which is comprised of hyphae and conidia and has a substantial amount of extracellular matrix [29]. *T. rubrum* biofilms exhibit a complex three-dimensional network with intricate branching patterns, as seen by scanning electron microscopy (SEM). The fungal hyphae and microconidia are encased in the biofilm’s intricate structure, which is abundant in extracellular polymeric substances (EPS), which increases their durability. The biofilm showed increasing density after a span of 72 hours. In Trichophyton rubrum biofilms, SEM pictures reveal that conidia are more noticeable than hyphae. This difference may be caused by various elements such as fungal characteristics, development stage, or environment, and it suggests active dispersal or reproduction. Additionally, the arrangement of the experiment may favor conidial development over hyphal growth [12]. The Trichophyton rubrum conidia are observed infecting the human epidermis using scanning electron microscopy. After two days, conidia germination and germ tube elongation are observed, and after four days, hyphae invasion and surface spread are observed [24]. The unique characteristics of conidia, the asexual reproductive spores of the dermatophyte *T. rubrum*, are visible in SEM pictures and are essential for recognizing fungal infections. Previous research employing SEM pictures revealed that applying anti-biofilm enzymes to the biofilm of V. parahaemolyticus breaks down the biofilm matrix and separates the majority of bacteria, indicating that matrix degradation is a successful anti-biofilm tactic [30]. A limitation of this study is the absence of chemical fixation and ethanol dehydration in SEM preparation, which may have influenced ultra-structural preservation. Future work will incorporate full fixation and quantitative image analysis to strengthen morphological interpretation.

FTIR spectroscopy revealed significant alterations in the chemical composition of *T.rubrum* biofilms after enzymatic treatment. Similar approaches have been used for fungal and bacterial biofilms. For instance [31] used ATR-FTIR to document biochemical shifts in polysaccharide and protein bands during Candida Albicans morphogenesis, supporting our interpretation of spectral changes after enzymatic treatment. FTIR analysis reveals the presence of exopolysaccharide components such as carbohydrates, proteins, and lipids. Examining the biofilm of T. rubrum and the cellulase enzyme-treated biofilm involves analyzing changes in beta 1–4 glycosidic bonds. Significant absorption bands for carbohydrates and glycosidic linkages typically appear between 1200 cm ⁻ ¹ and 900 cm ⁻ ¹ in FTIR spectra. In Fig 7 (control), key peaks are at 998.93 cm ⁻ ¹ and 1030.61 cm ⁻ ¹. In Fig 8 (cellulase-treated), a peak at 1026.88 cm ⁻ ¹ is noted, and the absence or shift of the 1030 cm ⁻ ¹ peak suggests that cellulase treatment might have altered the beta 1–4 glycosidic linkages, indicating structural changes in carbohydrates. FTIR spectra comparison between controls T. rubrum biofilm (Fig 7) and protease-treated T. rubrum biofilm (Fig 9) show distinct peak differences. The control biofilm exhibits peaks at 998.52654, 1036.30131, 1139.7437, 1205.79379, 451.79809, 1507.70816, 1636.30131, 19944.12574, 2126.44624, and 3334.0370 cm ⁻ ¹.The protease-treated biofilm lacks the 3334.10370 cm peak (indicative of O-H stretching), suggesting a change in the biofilm’s hydration or hydroxyl group content after treatment.

The FTIR spectral analysis of T. rubrum biofilms revealed significant differences between the untreated (Fig 7) and enzyme-treated (Fig 10) spectra. The untreated biofilm exhibits characteristic peaks associated with various molecular structures, including potential glycosidic bonds (998.92654 cm-1 and 1030.60891 cm-1) indicative of polysaccharides and an amide I band (1636.30131 cm-1) suggesting the presence of peptide bonds in proteins. In contrast, the biofilm treated with a combination of cellulase, protease, and amylase enzymes shows a distinctly different spectral profile. The absence of certain peaks and the appearance of new ones in the treated sample imply significant structural alterations in the biofilm composition. These changes likely reflect the enzymatic degradation of polysaccharides, proteins, and other biomolecules within the biofilm matrix. This comparative analysis provides valuable insights into the effectiveness of the enzyme combination in modifying the T. rubrum biofilm structure, which could have important implications for developing strategies to combat fungal infections and biofilm formation. Our FTIR analysis revealed marked reductions in amide I and II bands (\ ~ 1650 and \ ~ 1550 cm ⁻ ¹) as well as polysaccharide-associated signals (\ ~ 1200–1100 cm ⁻ ¹), consistent with prior studies using ATR‑FTIR to monitor fungal biofilm matrix degradation [32,33].

The present study demonstrates that cellulase, protease, and amylase enzymes exhibit significant inhibitory effects on mature Trichophyton rubrum biofilms, which are known for their structural resilience and resistance to antifungal agents. Similar to our findings, [13] highlighted the difficulty in eradicating mature dermatophyte biofilms due to enhanced extracellular polymeric substance (EPS) production and altered metabolic activity. Our results further validate the potential of enzymatic degradation strategies, aligning with prior reports that enzymes targeting EPS components can effectively destabilize fungal biofilm architecture [34]. Notably, cellulase, derived from T. rubrum, was particularly effective, suggesting that intra-species enzymatic targeting may enhance biofilm penetration and matrix dissolution.

From a clinical standpoint, these findings are highly relevant. Dermatophytoses, especially those caused by *T. rubrum*, often present as recurrent or chronic infections partly due to biofilm-mediated drug resistance [35]. The observed inhibition and eradication of biofilms by enzyme treatment opens avenues for combination therapies, where enzymes may be used as adjuncts to antifungals, improving drug access and efficacy. Such approaches could be especially beneficial in treating onychomycosis and tinea corporis, where biofilm-associated infections are notoriously difficult to manage. Further in vivo and formulation-based studies are warranted to translate these results into therapeutic interventions.

## 6. Conclusion

This study demonstrate that anti-biofilm enzymes—cellulase from *T.rubrum* and protease and amylase from *Candida Tropicalas* exhibited significant inhibitory effects on *T.rubrum* biofilms. Cellulase showed higher inhibition at the premature stage (64%), while combined enzymes achieved up to 60% eradication at the mature stage. The enzyme-treated groups showed substantial reduction in biofilm biomass, as confirmed by microtiter plate assay, SEM, and FTIR analyses. These findings indicate the potential of enzyme-based strategies as effective anti-biofilm approaches against dermatophyte infections, particularly in targeting early-stage biofilm development. Future studies should focus on in vivo validation and formulation development for clinical applications.
